# Artesunate inhibits osteoclastogenesis through the miR-503/RANK axis

**DOI:** 10.1042/BSR20194387

**Published:** 2020-07-21

**Authors:** Ming-Zhi Huang, Yong Zhuang, Xu Ning, Hao Zhang, Zhi-Min Shen, Xian-Wen Shang

**Affiliations:** Department of Orthopedics, the Affiliated Hospital of Guizhou Medical University, Guiyang 550004, P.R. China

**Keywords:** AKT pathway, Artesunate, MAPK pathway, miR-503, osteoclastogenesis, RANK

## Abstract

Osteoporosis is a metabolic bone disease that is characterized by decreased bone density and strength due to excessive loss of bone protein and mineral content, which can be induced by increased osteoclast activity. Developing agents targeting osteoclast activation is considered to be the most effective method to reverse bone destruction and alleviate the pain caused by osteoporosis. MTT assay was conducted to detect the cell viability after artesunate treatment of RAW264.7 cells. TRACP staining and pit formation assays were performed to examine the TRACP-positive cells and pit-forming activity of osteoclasts. qRT-PCR and Western blot analysis were performed to assess the mRNA and protein expression levels of the osteoclastogenesis-related genes NFATc1, TRAP, and cathepsin k. The protein levels of RANK, p-Akt, p-p38, and p-ERK were examined by Western blotting. Luciferase reporter assay was conducted to determine whether miR-503 targeted RANK directly. Artesunate inhibited TRACP-positive cells and the pit-forming activity of osteoclasts. However, artesunate increased the expression of miR-503. Artesunate suppressed osteoclastogenesis-related gene expression and RANKL-induced activation of MAPKs and the AKT pathway. In addition, miR-503 inhibited RANK expression by directly targeting RANK during osteoclast differentiation. Artesunate inhibited osteoclastogenesis and osteoclast functions *in vitro* by regulating the miR-503/RANK axis and suppressing the MAPK and AKT pathways, which resulted in decreased expression of osteoclastogenesis-related markers.

## Introduction

The bone remodelling process is characterized by a highly dynamic balance between bone absorption by osteoclasts (OC) and bone matrix synthesis by osteoblasts (OB). However, increased osteoclast activity could induce osteoporosis (OP), osteoarthritis (OA), rheumatoid arthritis (RA), periodontitis, and other lytic bone diseases [1–3]. OP is a systemic bone disease characterized by decreased bone mineral density (BMD) and disrupted skeletal architecture due to excessive loss of bone protein and minerals, predisposing the patient to increased fracture risks [4]. Postmenopausal osteoporosis (PMOP) is most common in primary osteoporosis, and the incidence has been rising rapidly [5]. Thus, agents that regulate the formation and function of abnormal OC are candidates for PMOP prevention and treatment.

Osteoclasts are differentiated from hematopoietic macrophage/monocyte lineage precursor cells in several stages, including proliferation, differentiation, fusion, and activation [6]. RANKL (receptor or activator of nuclear factor-kB NF-κB ligand) has been regarded as a major inducer of osteoclast differentiation [7]. During RANKL-induced osteoclastogenesis, the binding of RANKL to its receptor RANK (receptor activator of nuclear NF-κB) results in the recruitment and activation of adaptor TRAF6 and triggers the activation of MAP kinases (ERK, JNK and p38) and transcription factors such as NF-κB, AP-1, and NFAT [8,9]. The activation of these transcription factors can regulate osteoclast differentiation by promoting the expression of osteoclast-associated genes.

Artesunate is a semi-synthetic derivative of artemisinin, one of the most effective, low-toxicity drugs for clinical therapy of falciparum malaria [10]. In recent years, increasing studies have suggested that artesunate has an anti-inflammatory effect, suppressing NF-κB, IκB, TNF-α, and IL-6 to ameliorate colitis and sepsis and inhibit arthritis and adjuvant arthritis [11,12]. Furthermore, a recent paper by Wei et al. verified that artesunate could suppress osteoclastogenesis induced by RANKL and its bone resorption function, suggesting its value in the prevention and treatment of osteolytic diseases [13]. However, the underlying mechanism involved in the inhibitory effects of artesunate on osteoclastogenesis is not yet clear.

MicroRNAs (miRNAs) are a recently discovered class of small, noncoding RNAs of ∼22 nucleotides that negatively regulate gene expression at the post-transcriptional level. miR-503 was first identified in human retinoblastoma tissues using the miRNA microarray technique [14]. Several reports discovered the up-regulation of miR-503 in human parathyroid and adrenocortical carcinomas [15,16]. In addition, miR-503 was demonstrated to modulate the proliferation, migration, adhesion, and network formation of endothelial cells [17]. Besides, miR-503 was recently reported to regulate osteoclastogenesis by targeting RANK [18,19]. Moreover, several miRNAs have been reported to regulate the differentiation and function of osteoblasts and osteoclasts [18,20,21]. Christine et al. discovered that RBP-J-regulated miR-182 promoted osteoclastogenesis triggered by TNF-α [20]. miR-21 was reported to regulate osteoclastogenesis of bone marrow–derived monocyte/macrophage precursors (BMMs) by targeting programmed cell death protein 4 (PDCD4) [18,21]. Furthermore, new studies also revealed that artesunate suppressed cell proliferation and promoted cell apoptosis by regulating miRNAs in the development of different cancers [22,23]. However, whether miRNAs are involved in the inhibitory effects of osteoclastogenesis by artesunate remains unknown.

In this paper, we demonstrated that artesunate inhibited RANKL-induced osteoclastogenesis and osteoclast functions by regulating the miR-503/RANK axis and inhibiting the AKT and MAPK signaling pathways. Our results also indicated that artesunate may be effective in the treatment and prevention of osteoporosis.

## Materials and methods

### Cell culture and reagents

The murine monocytic cell line RAW264.7 was obtained from Shanghai Institute of Biochemistry and Cell Biology, Chinese Academy of Medical Sciences (Shanghai, China). RAW264.7 cells were cultured in a humidified incubator (5% CO_2_ in air) at 37 °C and maintained in DMEM containing 10% (v/v) heat-inactivated fetal calf serum (FCS) with 2 mM Gln, 100 units/ml penicillin, and 100 μg/ml streptomycin. Artesunate was purchased from Aladdin (Shanghai, China). The TRACP Staining Kit was purchased from Sigma (St. Louis, MO, U.S.A.). Mimic-miR-503 and inhibitor-miR-503 were purchased from GenePharma Co. Ltd. (Shanghai, China).

### MTT assay

The MTT staining assay was described previously by Mosmann [24] and was used with modifications. RAW264.7 cells were seeded in 96-well plates at 5 × 10^3^ cells per well and were treated with artesunate for 24, 48 or 72 h. After incubation, MTT solution was added into each well, and then the plate was incubated at 37°C for 4 h. Then, the supernatant was discarded, and the wells were washed with PBS three times. After drying, 200 μl of DMSO was added to each well. The plates were gently shaken to dissolve the formazan crystals. The absorbance was measured at 570 nm by a reference wavelength spectrophotometry of 630 nm using an ELX800 UV universal microplate reader (Bio-Tek Instruments Inc., Vermont, U.S.A.).

### TRACP staining assay

TRACP staining was performed in cultured RAW264.7 cells. RAW264.7 cells were seeded in six-well plates at 4 × 10^4^ cells per well. Cells were fixed with 4% formaldehyde in PBS and stained using a TRAP staining kit (Sigma-Aldrich, St. Louis, MO, U.S.A.) according to the manufacturer’s instructions. TRACP-positive cells with three or more nuclei were considered to be osteoclasts [25]. Cell images were obtained by a digital camera attached to an EVOS FL Auto microscope (Life Technologies, U.S.A.). ImageJ was used to count and analyze the number and areas of TRACP-positive multinucleated cells.

### Pit formation assay

RAW264.7 cells were plated at a density of 1.5 × 10^3^ cells/well on a bone biomimetic synthetic surface in 24-well plates (Corning, Corning, NY, U.S.A.) in the presence of 100 ng/ml RANKL (Sino biological, China) with or without artesunate (2, 4, or 8 μM). The medium was replaced on day 3 as described previously [26]. After 7 days, PBS was used to wash the plate, which was then dried for 5 h. The resorbed pits were observed using a light microscope (BX53; Olympus, Tokyo, Japan), and the resorbed area was analyzed using Image-Pro Plus software.

### qRT-PCR assay

RAW264.7 cells were plated in six-well plates at 4 × 10^4^ cells per well. Total RNA was isolated from RAW264.7 cells using TRIzol® reagent, and cDNA was synthesized using a reverse transcription reaction. Real-time PCR was performed using the SYBR Premix Ex Tag Kit (TaKaRa, Japan) according to the manufacturer’s instructions. The amplification conditions were as follows: 95°C for 10 min to activate the polymerization reaction, followed by 35 cycles of 10 s at 95°C, 15 s at 60°C, and 10 s at 72°C. The sequences of oligonucleotide primers for genes were presented in Table 1:

**Table 1:** Sequences of primers for genes

Primer	Sequence
GAPDH-F	5′-TGTTCGTCATGGGTGTGAAC-3′
GAPDH-R	5′-ATGGCATGGACTGTGGTCAT-3′
TRAP-F	5′-CACTCCCACCCTGAGATTTGT-3′
TRAP-R	5′-CCCCAGAGACATGATGAAGTCA-3′
NFATc1-F	5′-CAACGCCCTGACCACCGATAG-3′
NFATc1-R	5′-GGGAAGTCAGAAGTGGGTGGA-3′
Cathepsin K-F	5′-TGGGAGCTGTGGAAGAAGAC-3′
Cathepsin K-F	5′-TTTCATCCTGCCCCACATAC-3′
miR-503-F	5′-CCTATTTCCCATGATTCCTTCATA-3′
miR-503-R	5′-GTAATACGGTTATCCACGCG-3′
U6-F	5′- CTCGCTTCGGCAGCACA-3′
U6-R	5′- AACGCTTCACGAATTTGCGT-3′

### Western blotting analysis

RAW264.7 cells were plated in six-well plates at 4 × 10^4^ cells per well. RAW264.7 cells were treated with 8 μM artesunate or miR-503 mimics along with 100 ng/ml RANKL with or without miR-503 inhibitor for 5 days. Western blotting analysis was performed as previously reported [13]. β-Actin served as the loading control. Antibodies (1:1000) specific to p-ERK, p-P38, p-AKT, NFATc1, TRAP, cathepsin k, and β-actin were supplied by Cell Signaling Technology (Boston, U.S.A.). Secondary antibodies (1:5000) were supplied by Abcam (Cambridge, MA, U.S.A.).

### Luciferase assay

The procedure for the luciferase reporter assay has been previously reported [18,27]. RAW264.7 cells were plated in 96-well plates at 5 × 10^3^ cells per well. Cells were co-transfected using Lipofectamine 2000 with either WT or mutant pGL3-RANK constructs (200 ng) and miR-503 or miR-NC for 48 h. The Dual-Luciferase Reporter Assay System (Promega) was used to quantify luminescent signal by using a luminometer (Glomax, Promega). For each sample, the firefly luciferase activity value was normalized to the Renilla luciferase activity value from the co-transfected phRL-null vector (Promega).

### Statistical analysis

SPSS version 16.0 (IBM, Chicago, IL, U.S.A.) was used for statistical analysis. Data are presented as the mean ± standard deviation (SD) from three independent experiments, and significance was assessed with Student’s *t*-test. Differences were considered statistically significant when *P*<0.05 (*P<0.05, **P<0.01, and ***P<0.001).

## Results

### Artesunate impaired RANKL-induced osteoclastogenesis in RAW264.7 cells

**Figure 1:**
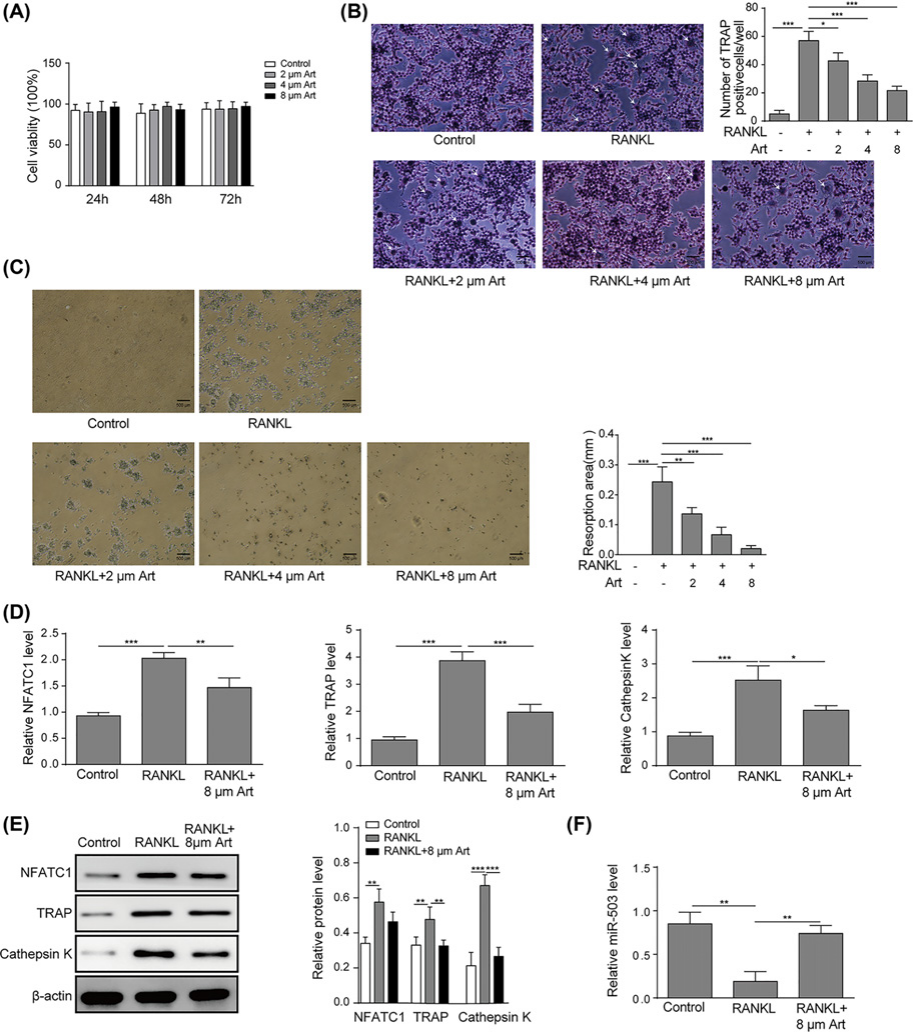
Artesunate impaired RANKL-induced osteoclastogenesis and inhibited cell viability in RAW264.7 cells (**A**) Cells were treated with artesunate at concentrations of 0, 2, 4, or 8 μM for 24, 48, or 72 h. Viability was detected by the MTT assay. Data from three independent experiments are presented. (**B**) RAW264.7 cells were treated with RANKL (100 ng/ml) and artesunate at concentrations of 0, 2, 4, or 8 μM, and the osteoclast differentiation assay was performed. After incubation, RAW264.7 cells were fixed, and the number of TRACP-positive multinucleated cells was counted; scale bars, 500 µm. The arrows in the diagram represent the formation of TRACP-positive osteoclasts. (**C**) RAW264.7 cells were seeded on hydroxyapatite-coated plates and treated with 0, 2, 4, or 8 μM concentrations of artesunate. Attached cells on the plates were removed and imaged under a light microscope (top); scale bars: 500 µm. (**D**) RAW264.7 cells were incubated with or without artesunate at a concentration of 8 μM, and the mRNA expression levels of NFATc1, TRAP and cathepsin k were detected by qRT-PCR. (**E**) RAW264.7 cells were treated with or without artesunate at a concentration of 8 μM, and the protein expression levels of NFATc1, TRAP, and cathepsin k were measured by Western blotting. (**F**) RAW264.7 cells were incubated with or without artesunate at a concentration of 8 μM, and the levels of miR-503 were detected by Western blotting. Data are presented as the mean±SD from three independent experiments; *P<0.05, **P<0.01, ***P<0.001.

To examine the effects of artesunate on cell proliferation, RAW264.7 cells were exposed to artesunate at concentrations of 2, 4, and 8 μM for 24, 48, and 72 h, and cell viability was detected using MTT assay. As shown in Figure 1A, artesunate exhibited few inhibitory effects on RAW264.7 cells. To investigate the influence of artesunate on osteoclastogenesis, RAW264.7 cells were incubated with RANKL at 2 μM, 4 μM or 8 μM concentrations of artesunate. As shown in Figure 1B, the total number of TRACP-positive cells in the artesunate-treated groups was significantly reduced compared with the RANKL group, and the number of TRACP-positive cells in the 8 μM group was dramatically less than that in the 2 μM group. Then, we investigated whether osteoclastic bone resorption could be inhibited by artesunate *in vitro*. A pit-formation assay was conducted to examine the effects of artesunate on the pit-forming activity of cells on a bone biomimetic synthetic surface. The results showed that artesunate treatment inhibited osteoclast activity, as suggested by the impaired pit-forming activity of osteoclasts. After the osteoclast preparation was cultured for 24 h, a number of resorption pits formed on the plates. Artesunate added at 2, 4 or 8 μM obviously inhibited the formation of resorption pits by osteoclasts (Figure 1C). Taken together, these findings demonstrated that artesunate inhibited RANKL-induced osteoclastogenesis and osteoclast functions in RAW264.7 cells in a dose-dependent manner.

RANKL-induced osteoclastogenesis resulted in changes in related marker genes, such as TRAP and cathepsin K, which are downstream genes of NFATc1 [25,28]. NFATc1 is considered a major regulator of osteoclastogenesis and function. We further examined the influence of artesunate on the expression of the above genes during osteoclastogenesis. The results of qRT-PCR and Western blot analysis showed that after incubation with RANKL, both mRNA and protein expression levels of NFATc1, TRAP, and cathepsin k were dramatically inhibited by artesunate at a concentration of 8 μM (Figure 1D,E). RANKL significantly induced the inhibition of miR-503 expression, while artesunate abolished this inhibitory effect (Figure 1F). Therefore, artesunate treatment significantly recovered the reduced miR-503 levels induced by RANKL and suppressed osteoclastogenesis by impairing osteoclastic-specific genes. These results showed that artesunate impaired RANKL-triggered osteoclastogenesis in RAW264.7 cells.

### Artesunate inhibited RANKL-induced activation of MAPKs and the AKT pathway

**Figure 2:**
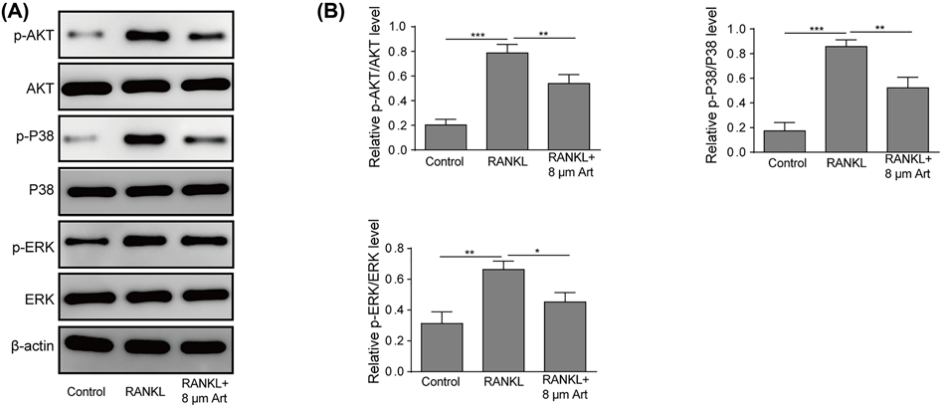
Artesunate inhibited RANKL-induced activation of MAPKs and the AKT pathway (**A** and **B**) The phosphorylation of AKT, p38 and ERK protein was inhibited by Artesunate at a concentration of 8 μM. Data are presented as the mean ± SD from three independent experiments; *P<0.05, **P<0.01.

RANKL interacts with RANK on osteoclast precursor cells, subsequently leading to the activation of downstream pathways, during which MAPKs and AKT play important roles in osteoclastogenesis. To evaluate the influence of artesunate on MAPKs and the AKT pathway after treatment with RANKL, we detected p-Akt, p-p38, and p-ERK protein expression by Western blot analysis. In the present study, RAW264.7 cells were divided into three groups: a control group, RAW264.7 cells with RANKL, and RAW264.7 cells with RANKL and incubated with 8 μM artesunate. These data suggested that artesunate suppressed the phosphorylation of the AKT, p38, and ERK proteins (Figure 2A,B), indicating inhibitory effects on MAPKs and the AKT pathway.

### miR-503 inhibited RANK expression by directly targeting RANK during osteoclast differentiation

**Figure 3:**
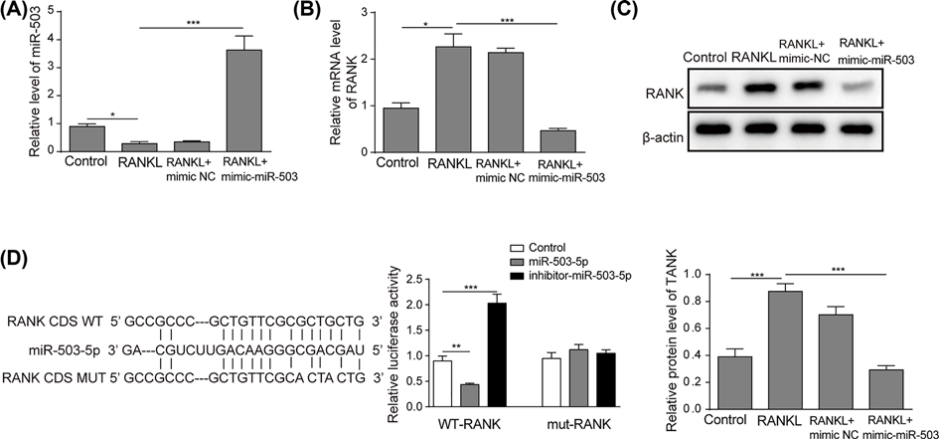
miR-503 inhibited RANK expression by directly targeting RANK during osteoclast differentiation Cells were incubated with RANKL (100 ng/ml) and transfected with or without mimic-miR-503. (**A**) The mRNA expression level of miR-503 was detected by qRT-PCR. (**B**) RANK mRNA expression was detected by qRT-PCR. (**C**) RANK protein expression was measured by Western blotting. (**D**) Cells were co-transfected with the luciferase reporter carrying WT-pGL3-RANK or MUT-pGL3-RANK, phRL-null, and miR-NC or miR-503. Luciferase activity was detected after 48 h of transfection. Data are presented as the mean ± SD from three independent experiments; *P<0.05, **P<0.01.

Recently, miR-503 has been reported to directly target RANK [18], which is activated by the interaction of RANKL to promote osteoclast differentiation. Therefore, we hypothesized that artesunate may inhibit osteoclastogenesis through the miR-503/RANK axis. First, to define the expression of miR-503 and RANK during osteoclast differentiation, we validated the expression of miR-503 and RANK in cells treated with RANKL. As shown in Figure 3A–C, miR-503 expression was notably reduced, while the mRNA and protein levels of RANK were significantly elevated during osteoclast differentiation induced by RANKL stimulation. However, the effects induced by RANKL were dramatically reversed by mimic-miR-503 (Figure 3A–C). To confirm whether miR-503 directly bound to RANK to regulate osteoclastogenesis, a luciferase expression vector carrying the CDS of RANK (WT-pGL3-RANK) was co-transfected with miR-503 into RAW264.7 cells treated with RANKL and the effect of miR-503 on luciferase translation was detected using the level of luciferase activity. As shown in Figure 3D, miR-503 overexpression inhibited the luciferase activity of the RANK CDS reporter gene. However, the mutation of two nucleotides within the sequences of the putative target site in the CDS of RANK (MUT-pGL3-RANK) reversed this suppression, confirming the specificity of the effect. In conclusion, these results suggested that miR-503 inhibited RANK expression by directly targeting RANK in RANKL-induced osteoclast differentiation.

### miR-503 suppressed RANKL-induced osteoclastogenesis and osteoclast functions

**Figure 4:**
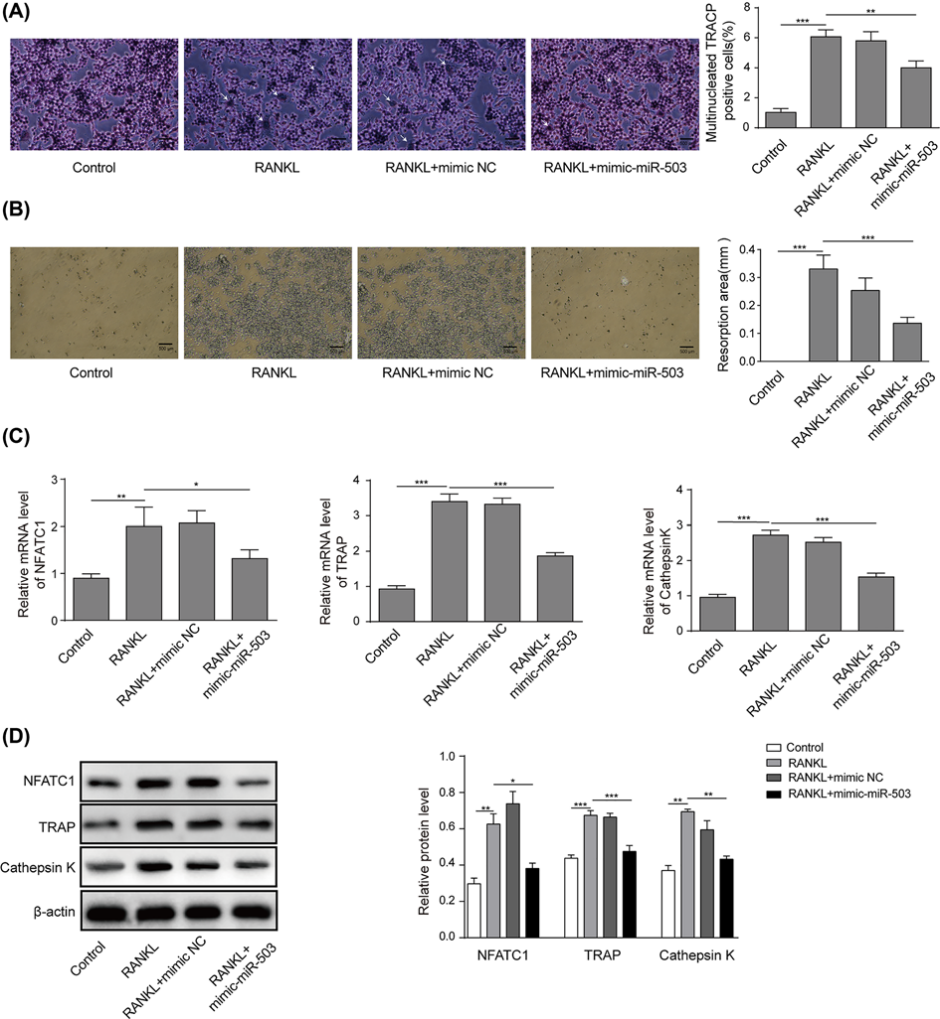
miR-503 suppressed RANKL-induced osteoclastogenesis and osteoclast function RAW264.7 cells were incubated with RANKL (100 ng/ml) and transfected with or without mimic-miR-503. (**A**) The osteoclast differentiation assay was performed. The number of TRACP-positive multinucleated cells was counted; scale bars: 500 µm. The arrows in the diagram represent the formation of TRACP-positive osteoclasts. (**B**) Cells were plated on hydroxyapatite-coated plates, and the cells attached on the plates were removed and imaged under a light microscope (top). Scale bars: 500 µm. (**C** and **D**) The mRNA and protein expression levels of NFATc1, TRAP, and cathepsin k were detected by qRT-PCR assay and Western blot assay, respectively. Data are presented as the mean ± SD from three independent experiments; *P<0.05, **P<0.01.

To further investigate the effects of miR-503 on osteoclastogenesis and osteoclast functions, we first conducted a TRACP staining assay in RAW264.7 cells transfected with or without mimic-miR-503. As shown in Figure 4A, RANKL incubation obviously increased the total number of TRACP-positive cells, while overexpression of miR-503 suppressed the TRACP-positive cells induced by RANKL. We further explored whether miR-503 suppresses osteoclastic bone resorption *in vitro*. Consistent with the above findings, the results of the pit-formation assay showed that osteoclast activity was severely impaired by mimic-miR-503 transfection, as demonstrated by the decreased formation of resorption pits by osteoclasts, suggesting that miR-503 significantly inhibited the pit-forming activity of osteoclasts, which was enhanced by RANKL (Figure 4B). Then, qRT-PCR and Western blot assay were performed to examine the effects of miR-503 on the mRNA and protein levels of osteoclastic-specific genes. As shown in Figure 4C,D, treatment with mimic-miR-503 reversed the enhancement of NFATc1, TRAP and cathepsin k expression induced by RANKL. Our data demonstrated that miR-503 suppressed RANKL-induced osteoclastogenesis and osteoclast functions.

### miR-503 inhibited RANKL-triggered activation of MAPKs and the AKT pathway

**Figure 5:**
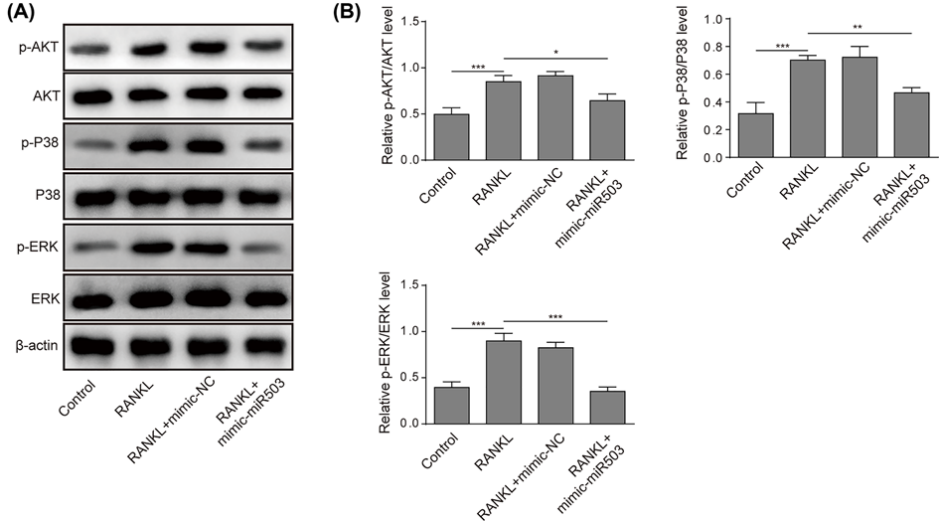
miR-503 inhibited RANKL-induced activation of MAPKs and the AKT pathway RAW264.7 cells were incubated with RANKL (100 ng/ml) and transfected with or without mimic-miR-503. (**A** and **B**) The protein expression of p-AKT, p-p38, and p-ERK was measured by Western blotting. Data are presented as the mean ± SD from three independent experiments, *P<0.05, **P<0.01.

To validate the influence of miR-503 on MAPKs and the AKT pathway after treatment with RANKL, we examined the protein expression of p-Akt, p-p38, and p-ERK by western blot analysis. As shown in Figure 5A,B, RANKL stimulation dramatically increased the expression of p-Akt, p-p38, and p-ERK, which was then inhibited by mimic-miR-503 transfection into RAW264.7 cells. The results illustrated that miR-503 inhibited the activation of MAPKs and the AKT pathway induced by RANKL.

### Artesunate inhibited osteoclastogenesis through the miR-503/RANK axis

**Figure 6:**
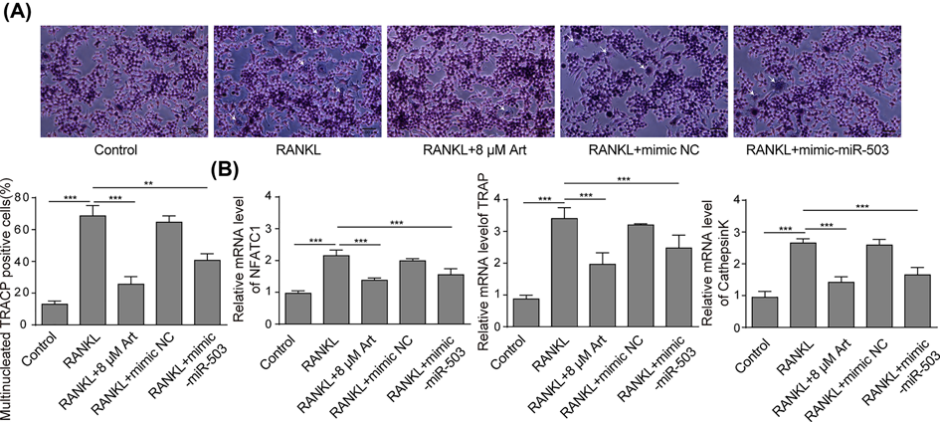
Artesunate inhibited osteoclastogenesis through the miR-503/RANK axis RAW264.7 cells were treated with RANKL (100 ng/ml) in the presence of 8 μM artesunate with or without miR-503 inhibitor. (**A**) The osteoclast differentiation assay was performed. The number of TRACP-positive multinucleated cells was counted; Scale bars: 500 µm. The arrows in the diagram represent the formation of TRACP-positive osteoclasts. (**B**) The mRNA expression levels of NFATc1, TRAP, and cathepsin k were measured by qRT-PCR. Data are presented as the mean ± SD from three independent experiments; *P<0.05, **P<0.01, ***P<0.001.

Since the above findings suggested that artesunate impaired RANKL-induced osteoclastogenesis and that miR-503 overexpression suppressed osteoclastogenesis by targeting RANK, we hypothesized that artesunate may inhibit osteoclastogenesis through the miR-503/RANK axis. First, the TRACP staining assay was performed to test osteoclastogenesis in RAW264.7 cells. The results showed that the total number of TRACP-positive cells in the artesunate-treated groups was significantly decreased compared with the control group, while treatment with the miR-503 inhibitor reversed the decrease in TRACP-positive cells induced by artesunate (Figure 6A). Furthermore, the results of qRT-PCR assay indicated that the decrease in miR-503 by the inhibitor remarkably elevated the expression of NFATc1, TRAP, and cathepsin k mRNA, which was inhibited by artesunate (Figure 6B). These results indicated that artesunate inhibited osteoclastogenesis through the miR-503/RANK axis.

## Discussion

In the late 1990s, Horwood et al. found that activated T cells participated in the loss of bone protein and mineral content by inducing the differentiation and maturation of bone resorption cells (osteoclasts) *in vitro* [29]. Arron et al. first proposed the concept of osteoimmunology in 2000 and reported the interaction between the skeletal system and the immune system. Osteoimmunology-related diseases, such as osteoporosis and inflammatory arthritis, are characterized by excessive osteoclast activation. Activation of osteoclasts can cause bone resorption, bone loss, and even trigger bone fractures. Drugs that target osteoclasts can be used to treat osteoporosis. Currently, bisphosphonates (BPs) are considered the most important anti-resorptive drugs for bone disease therapy. However, their application is limited by side effects such as osteonecrosis. Emerging therapy with biological drugs such as denosumab are too expensive for the public, although denosumab exhibited potent effects. Thus, novel strategies should be explored to effectively and safely target osteoclasts.

Artesunate is a semi-synthetic derivative of artemisinin, generally considered an antimalarial drug. In recent years, increasing studies have suggested that artesunate has an anti-inflammatory effect. Jiang et al. reported that artesunate impaired atherosclerosis lesion formation alone or in combination with rosuvastatin by inhibiting pro-inflammatory cytokines and pro-inflammatory chemokines [30]. Guruprasad et al. found that artesunate improved functional outcomes in rats by maintaining oxidative homeostasis and suppressing the expression of COX-2 [31]. In addition, artesunate was reported to inhibit cigarette smoke and ovalbumin concurrent exposure-triggered airway inflammation and might repair glucocorticoid insensitivity [32]. Besides, artesunate could suppress osteoclastogenesis induced by RANKL and its bone resorption function *in vivo* [13]. In the present study, we found that artesunate is an inhibitor of osteoclast differentiation and function. Artesunate suppressed RANKL-induced osteoclastogenesis and osteoclast functions in RAW264.7 cells in a dose-dependent manner, with inhibitory effects on the pit-forming activity of osteoclasts. Compared with the current drugs for bone disease therapy, artesunate has fewer side effects and is less expensive. The *in vivo* experiment is needed to demonstrate the utility of artesunate, we will conduct the in vivo experiment in the further study.

RANKL is considered a major inducer of osteoclast differentiation. Macrophage colony-stimulating factor (M-CSF), interleukin-1 (IL-1), tumor necrosis factor alpha (TNF-α), and other cytokines and factors act as additional enhancers [33,34]. Binding of RANKL with its receptor RANK can activate and accumulate the adaptor protein TRAF6-TAK1 complexes, which results in the activation of the NF-κB and MAPK pathways, subsequently leading to increased expression of transcription factors c-Fos and NFATc1. The activation of the above pathways consequently influences several osteoclastogenesis-related marker genes, such as TRAP, MMP-9, and cathepsin K, which results in the formation of bone resorption pits during osteoclast differentiation. Targeting the above signaling pathways and regulation of osteoclastogenesis-related genes may be beneficial for osteoporosis therapy. In the present study, artesunate suppressed the RANKL-induced expression of OC marker genes in RAW264.7 cells, with a sharp decrease in the expression of NFATc1, c-Fos, MMP-9, TRAP, and cathepsin k.

RANKL binds RANK on osteoclast precursor cells, subsequently leading to the activation of downstream pathways, during which NF-κB, MAPKs, and AKT play important roles in signal transduction [35–37]. In response to RANKL, RANK interacts with TRAFs, among which TRAF6 is crucial for the activation of MAPKs [38]. Then, TRAF6 induces the activation of the TAB1/TAB2/TAK1 complex and activates the expression of IKK-β and MAPKs [9]. Besides, RANK also causes the activation of Src family kinase signalling, subsequently activating AKT by binding with the TRAF6 and Cbl scaffolding proteins [9]. In addition, Zhi et al. reported that l-tetrahydropalmatine suppressed osteoclastogenesis *in vivo* and *in vitro* via blocking RANK–TRAF6 interactions and inhibiting NF-κB and MAPK pathways [39]. In the present study, we demonstrated that artesunate inhibited the MAPK pathway and AKT pathway, including p-AKT, p-ERK, and p-p38, which led to decreased expression of osteoclastogenesis-related markers, including NFATc1, TRAP, and cathepsin k.

Recently, miRNAs have been reported to be involved in modulating the differentiation and function of osteoblasts and osteoclasts [18,20,21]. Miller et al. demonstrated that RBP-J-regulated miR-182 promoted osteoclastogenesis induced by TNF-α [20]. miR-21 was also reported to regulate osteoclastogenesis of bone marrow-derived monocyte/macrophage precursors (BMMs) by targeting programmed cell death protein 4 (PDCD4) [18,21]. A previous study revealed that miR-503 regulated osteoclastogenesis by targeting RANK [18]. Chen et al. confirmed that RANK is a target of miR-503 and induced osteoclast differentiation. Furthermore, miR‐503 regulated M-CSF^ +^ RANKL-induced osteoclastogenesis of CD14^+^ PBMCs by targeting RANK. In the present study, we also demonstrated that miR-503 suppressed RANKL-induced osteoclastogenesis and bone resorption and inhibited the activation of MAPKs and the AKT pathway.

In summary, we revealed for the first time that artesunate inhibited RANKL-induced osteoclastogenesis and osteoclast functions by regulating the miR-503/RANK axis and inhibiting the AKT and MAPK signalling pathways. This research provides a new molecular mechanism for the treatment of bone diseases and suggests that artesunate could serve as an effective alternative agent for the prevention and therapy of osteoclast-related disorders.

## Data Availability

All data generated or analyzed during this study are included in this paper.

## Abbreviations

BMD: bone mineral density
BMM: bone marrow–derived monocyte/macrophage
OA: osteoarthritis
OB: osteoblasts
OC: osteoclasts
PDCD4: programmed cell death protein 4
PMOP: postmenopausal osteoporosis
RANKL: receptor or activator of nuclear factor-kB NF-κB ligand
